# The 3d left ventricular geometry integrated in myocardial wall stress estimation is more sensitive than end diastolic mass/volume ratio to characterize afterload-related left ventricular remodeling

**DOI:** 10.1186/1532-429X-13-S1-P8

**Published:** 2011-02-02

**Authors:** Nadjia Kachenoura, Marion Sénési, Carine Defrance, Emilie Bollache, Ludivine Perdrix, Alban Redheuil, Elie Mousseaux

**Affiliations:** 1 INSERM U678, Paris, France; 2 ETH, Zurich, Switzerland; 3 Echocardiography Department HEGP, Paris, France; 4 Radiology department, Paris, France

## Purpose

To analyze left ventricular (LV) remodeling using an accurate 3D evaluation of afterload-related changes in LV geometry.

## Introduction

To maintain an effective LV-arterial coupling the LV adapts to the increased afterload caused by aging or cardiovascular disease. However, subsequent changes in LV mass and concentric remodeling have been associated with poor outcome. To understand LV remodeling, we studied variations of 3D myocardial wall stress (MWS), its geometrical factor as well as diastolic LV mass to volume ratio (LVM/EDV) on a population with a wide range of afterload.

## Methods

Indeed, we studied 57 patients divided into three subgroups: 1) C1 included 22 healthy subjects aged between 22 and 37 years (26±5 years), (2) C2 included 23 healthy subjects aged between 41 et 81 years (55±9 years) and 3) AVS included 12 subjects (75±14 years) with aortic valve stenosis (AVS) characterized by (valve area=0.78±0.19 cm^2^). All subjects had short axis cine CMR acquisitions (GE 1.5T) followed by carotid applanation tonometry calibrated using brachial pressures recorded during CMR. After myocardial delineation, the LV geometrical factor LV_GF_, previously described by Grossman, was calculated as a combination of the local LV radius and myocardial thickness while considering the LV longitudinal curvature to correct for partial volume effects, especially in apical slices. This LV_GF_ was combined with peak systolic pressures (PSP) resulting in MWS. For AVS patients, the echocardiographic transaortic valve maximal gradient was added to the tonometric PSP.

## Results

**Figure 1:**
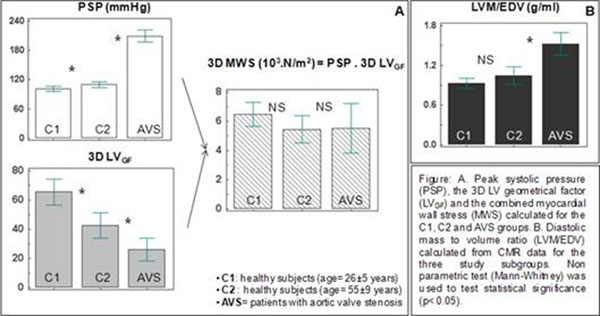
A. Peak systolic pressure (PSP), the 3D LV geometrical factor (LV_GF_) and the combined myocardial wall stress (MWS) calculated for the C1, C2 and AVS groups. B. Diastolic mass to volume ratio (LVM/EDV) calculated from CMR data for the three study subgroups. Non parametric test (Mann-Whitney) was used to test statistical significance (p<0.05).

Ejection fraction was homogeneous between the three subgroups (C1=64±5, C2=66±7, AVS=72±8 %) The significant and gradual elevation in PSP found between the three groups (C1=101±12, C2=110±13, AVS=209±20 mmHg) caused changes in LV geometry (figure 1), which were well characterized by the 3D LV_GF_ but only partially by LVM/EDV. Indeed, gradual and significant changes in LV_GF_ were found for the three groups (C1=65±20, C2=42±19, AVS=26±12) while LVM/EDV (C1=0.92±0.18, C2=1.04±0.30, AVS=1.52±0.27 g/ml) was increased in AVS significantly but failed to discriminate between age groups without AVS. Gradual changes in LV geometry reflected by the 3D LV_GF_ demonstrated the ability of the LV to adapt to the increased afterload and therefore to maintain constant MWS. Indeed, no significant variations in MWS were found between the three subgroups (C1=6.5±1.9, C2=5.4±2.1, AVS=5.5±2.6 10^3^.N/m^2^)

## Conclusions

The described 3D evaluation of LV geometry, which can be easily integrated to standard CMR LV function evaluation since it only requires routine myocardial delineation in systole, sensitively characterized LV remodeling related to aging or to AVS.

